# The association of mitochondrial DNA haplotypes and phenotypic traits in pigs

**DOI:** 10.1186/s12863-018-0629-4

**Published:** 2018-07-06

**Authors:** Justin C. St. John, Te-Sha Tsai

**Affiliations:** Centre for Genetic Diseases, Hudson Institute of Medical Research and Department of Molecular and Translational Science, Monash University, 27-31 Wright Street, Clayton, Vic 3168 Australia

**Keywords:** Mitochondrial DNA, Haplotype, Pig, Phenotype, Maternally inherited, Evolutionary trade offs

## Abstract

**Background:**

The mitochondrial genome (mtDNA) is an emerging determiner of phenotypic traits and disease. mtDNA is inherited in a strict maternal fashion from the population of mitochondria present in the egg at fertilisation. Individuals are assigned to mtDNA haplotypes and those with sequences that cluster closely have common origins and their migration patterns can be mapped. Previously, we identified five mtDNA haplotypes in the commercial breeding lines of Australian pigs, which defined their common origins, and showed how these mtDNA haplotypes influenced litter size and reproductive function in terms of egg and embryo quality and fertilisation efficiency.

**Results:**

We have determined whether mtDNA haplotypes influence other phenotypic traits. These include fat density; muscle depth; fat to leanness ratios; lifetime daily gain; teat quality; muscle score; front and rear leg assessments; percentage offspring weaned; weaning to oestrus intervals; gilt age at selection; and gestational length. In all, we assessed 5687 pigs of which 2762 were females and 2925 were males. We assessed all animals together and then by gender. We further assessed by gender based on whether a sire had joined with females from only one haplotype or from more than one haplotype. We determined that fat density, muscle depth, fat to leanness ratios, lifetime daily gain and teat quality were influenced by mtDNA haplotype and that there were gender specific effects on teat quality.

**Conclusions:**

Our data illustrate that mtDNA haplotypes are associated with a number of important phenotypic traits indicative of economic breeding values in breeding pigs with gender-specific differences. Interestingly, there are ‘trade offs’ whereby some mtDNA haplotypes perform better for one selection criterion, such as muscle depth, but less so for another, for example teat quality, indicating that pig mtDNA haplotypes are afforded an advantage in one respect but a disadvantage in another.

**Electronic supplementary material:**

The online version of this article (10.1186/s12863-018-0629-4) contains supplementary material, which is available to authorized users.

## Background

The maternally inherited mitochondrial genome is double stranded and, in the pig, is approximately 16.7 kb in size [1]. It encodes 13 subunits of the electron transfer chain, 22 tRNAs and 2 rRNAs. It also possesses one major non-coding region, the D-loop, which is the site of interaction for the nuclear-encoded transcription and replication factors that translocate to the mitochondrion to initiate transcription and then replication of the mitochondrial genome. The D-loop also possesses two hypervariable regions (HVI and HVII), which are frequently used by molecular geneticists to identify the maternal ancestral patterns of mitochondrial DNA (mtDNA) transmission and the migration patterns of individuals worldwide [2].

Over time, maternal lineages have evolved as individuals and their mitochondrial genomes have adapted to environmental changes [2]. As the resultant mutations have become fixed, and different mitochondrial lineages have arisen from one or more common origins, mitochondrial genomes are characterized by their clustering into groupings known as mtDNA haplotypes [3]. Consequently, mtDNA haplotypes have been able to distinguish between breeds and strains of organisms and conferred advantages and disadvantages to the organism [3]. In humans, mtDNA haplotypes have been associated with a number of diseases such as cancer [4], diabetes [5], Alzheimer’s [6] and Parkinson’s [7]. In cattle, there have been associations with calving rates [8], meat [9] and milk [10] quality traits, embryo production efficiency [11, 12], and body weight in, for example, American bison carrying cattle mtDNA [13]. In pigs, there are associations with body weight [14] and meat quality [15]. Furthermore, associations have been reported with reproductive capacity and litter size in pigs [16].

**Fig. 1:**
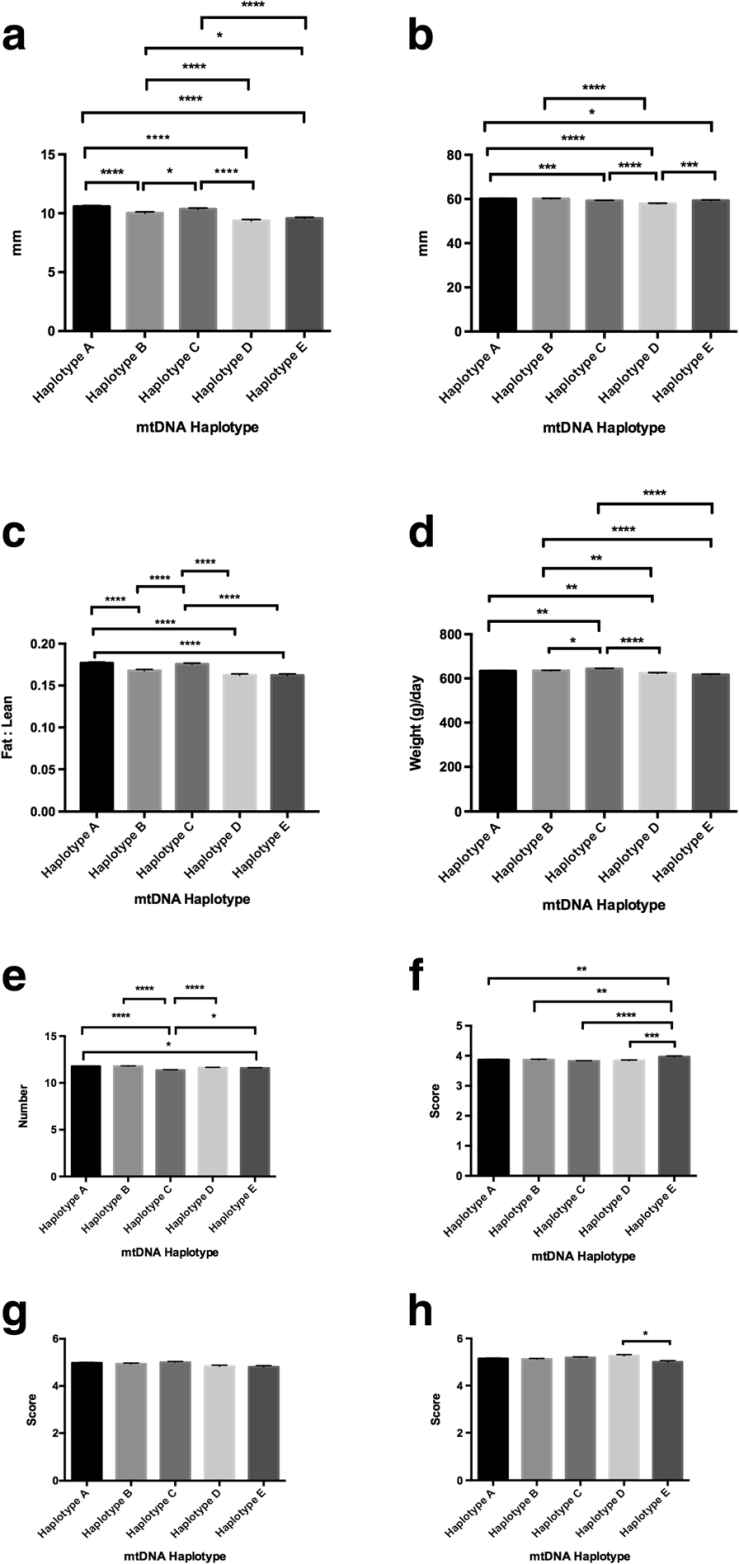
Assessment of phenotypic traits relative to mtDNA haplotype for all animals investigated. Graphs represent mean ± SEM values for (**a**) fat density; (**b**) muscle depth; (**c**) fat to leanness ratio; (**d**) lifetime daily gain; (**e**) good teat quality; (**f**) muscle score; (**g**) front leg score; and (**h**) rear leg score. * = *P* < 0.05; ** = *P* < 0.01; *** = *P* < 0.001; **** = *P* < 0.0001

**Fig. 2:**
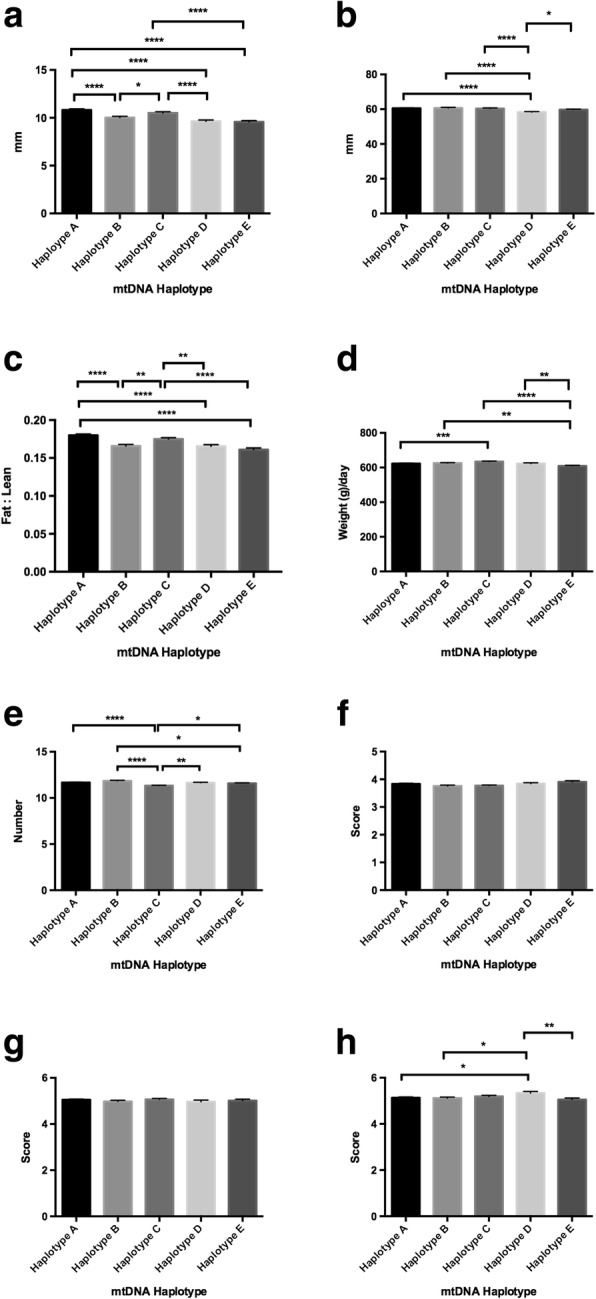
Assessment of phenotypic traits relative to mtDNA haplotype for all females investigated. Graphs represent mean ± SEM values for (**a**) fat density; (**b**) muscle depth; (**c**) fat to leanness ratios; (**d**) lifetime daily gain; (**e**) good teat quality; (**f**) muscle score; (**g**) front leg score; and (**h**) rear leg score. * = *P* < 0.05; ** = *P* < 0.01; *** = *P* < 0.001; **** = *P* < 0.0001

Amongst Australian commercial pigs, our previous study identified five mtDNA haplotypes, three of which cluster with pigs of Asian origin and two with European pigs [16] (as shown in Figs. 1 and 2 of reference [16]), despite all demonstrating phenotypes that are typically European in origin. These five haplotypes exhibit distinct reproductive capacities, which influence litter size. Indeed, each of the haplotypes exhibits a distinct level of developmental competence in terms of oocyte quality, which determines the oocyte’s ability to fertilise and develop to the blastocyst stage, the final stage of preimplantation development, and, thus, the availability of embryos to implant and give rise to live offspring.

Nevertheless, there are a number of traits that are important to pig production, which are collectively defined as estimated breeding values [17]. Estimated breeding values allow an animal to be assessed for overall production efficiency and this is important information to the breeding and meat producing industries. Key criteria include reproductive factors, such as number of live born, litter size and teat quality; factors related to meat quality, for example, fat density and muscle depth; and lifetime daily gain, namely assessment of weight gained between birth and death. These criteria will determine whether it is feasible to maintain lines of animals in breeding programs and how the breeding programs will be managed [18]. However, the genetic assessment of these traits is primarily based on the analysis of polymorphisms within genes located on the chromosomal genome [17] and often neglects the mitochondrial genome. Indeed, studies in cell lines, where cells possess the same chromosomal DNA but different mtDNA haplotypes, demonstrated that mtDNA haplotypes influenced chromosomal gene expression patterns [19] and the cell’s metabolic activity [20].

As mtDNA haplotypes are associated with adaptation to environment, disease and reproductive functions, we set out to determine whether other specific performance traits associated with pigs, which are indicative of their estimated breeding values, would be influenced by their mtDNA haplotypes, and specifically whether those haplotypes with low litter size may have traded reproductive capacity in order to promote other specific traits. In this study, we assessed fat density, muscle depth, fat to leanness ratios, lifetime daily gain, teat quality, muscle and leg scores, percentage offspring weaned, weaning to oestrus intervals, gilt age at selection, and gestational length for pigs from each of the five haplotypes to determine whether their mtDNA haplotypes are associated with these traits over multiple generations. It is evident that there is a trade-off between litter size and reproductive capacity and some of these other key traits.

## Methods

### Environment and data

Data were obtained from 5687 pigs of which 2762 were females and 2925 were males (see Additional file 1: Table S1), which spanned 18 breeds/lines (Additional file 2: Table S2). All pigs were raised on one farm under similar environmental conditions and were direct maternal descendants and ancestors of the 94 sows analysed in [16] (as shown in Figs. 1 and 2 of reference [16]), which were assigned to five mtDNA haplotypes (A to E; Additional file 3: Figure S1). No tissue material was collected from live animals for these experiments. Data from each animal were obtained from an Australian Pork Ltd. database with permission from Australian Pork Ltd. to analyse the data collected.

### Haplogroup assignment and evolutionary divergence analysis

Haplogroup assignment was performed using the MitoToolPy application [21] using whole mtDNA sequences previously deposited in the NCBI database (https://blast.ncbi.nlm.nih.gov) [16], namely mtDNA haplotypes A (KT279758.1), B (KT261429.1), C (KT279759.1), D (KT279760.1) and E (KT261430.1). Evolutionary divergence analysis was conducted in MEGA6 [22] in order to determine relationships amongst the pig mtDNA haplotypes. Whole mtDNA sequences were firstly aligned using ClustalW within the MEGA6 software. Model testing using the Bayesian Information Criterion (BIC) score was then performed to determine the most appropriate model for Maximum Likelihood tree construction. The Maximum Likelihood phylogenetic tree was constructed by applying the Neighbor-Joining method to a matrix of pairwise distances estimated using the Maximum Composite Likelihood (MCL) approach. The tree topology was analysed based on the General Time Reversible model [23, 24] supported by 1000 bootstrap replicates. The timetree shown was generated using the RelTime method [25], based on the European and Asian split of 750,000 years before present (YBP) [26].

### Assessment of fat density and muscle depth

Ultrasonic backfat depth and loin muscle area were determined from a cross sectional or transverse image of the loin muscle using an Aloka SSD 500 V scanner fitted with a 12.45 cm probe. The measurements were taken at the interface of the 10th and 11th ribs. Data were collected and assessed using the BioSoft Toolbox® II for Swine at day 153 or as close to and at a weight as close to 100 kg. Measurements were recorded in mm. These data were then expressed as ratios to determine fat to leanness ratios but did not include weight values as these were only approximate at time of data collection.

### Determination of lifetime daily gain

Lifetime daily gain was assessed by recording the animal’s endpoint weight and dividing by the number of days alive.

### Teat quality

Teat quality was assessed visually for the left side of each animal and the number of good (functional) teats recorded.

### Assessment of muscle and front and rear legs

Muscle and front and rear legs were visually assessed and assigned a score. Specifically, muscle quality was assessed by determining rump definition where a score of 1 was lowest and 10 highest; front and rear legs were assessed for conformation, straightness, flexibility and gait. For rear legs, a score of 5 was considered highest, with variance either side of 5 being indicative of poorer quality. For front legs, animals were assigned a score of 1 (lowest) to 10 (highest).

### Percentage offspring weaned

The percentage offspring weaned from each parity was determined from the number of liveborn animals that survived through to weaning per parity and calculated as: (No. weaned / No. liveborn) X 100.

### Weaning to oestrus intervals

The weaning to oestrus interval was determined as the interval between the last day of lactation and the first day of insemination. The interval period was expressed in days.

### Gilt age at selection

The age of each gilt at selection was determined from the date of the first insemination and expressed in days.

### Length of gestation

The length of gestation was determined from the first day of insemination to the last day of pregnancy (delivery date) and was expressed in days.

### Statistical analysis

All statistical analyses were performed using GraphPad Prism version 6.0d for Mac (GraphPad Software, La Jolla, California, USA). Data were tested for normality, and those that followed Gaussian distribution were analysed using one-way ANOVA followed by post-hoc multiple comparisons. Those that did not follow Gaussian distribution were analysed using the Kruskal-Wallis test followed by post-hoc multiple comparisons.

## Results

Having previously identified five mitochondrial DNA haplotypes within a commercial pig breeding population in Australia [16] (specifically, Figs. 1 and 2 of reference 16; and Additional file 3: Figure S1), we further assigned them to their respective haplogroups using the MitoToolPy application [21] to provide further identification of pigs used in this work. We found that mtDNA haplotype A is equivalent to D1B; haplotype B to D1e’g’h; haplotype C to D3a; haplotype D to E1a1a; and haplotype E to E. We then determined the time of divergence between each haplotype. The estimated divergence time for mtDNA haplotypes A and B was 50,000 YBP; A and C 90,000 YBP; A and D 750,000 YBP; and D and E 50,000 YBP (Additional file 3: Figure S1). Therefore, each mtDNA haplotype is from distinct maternal lineages and has evolved over thousands of years.

Having established the genetic divergence between each of the haplotypes, we assessed pigs from each haplotype for the measurement of fat density and muscle depth by ultrasound; lifetime daily gain based on weight gain from birth to death; and based on visual appraisal of muscle, front leg and rear leg scores and the number of good teats. All data sets were collected from one farm, and, as mtDNA is solely maternally inherited, we included maternal ancestors and descendants of each female (range = 4 to 10 generations), which was previously assigned to a specific mtDNA haplotype based on Sanger and Next Generation sequencing [16]. Additional file 1: Table S1 shows the total number of animals analysed for each haplotype; a breakdown of the number of males and females associated with each haplotype; and the number of sires that serviced the females to produce the offspring for which the data are presented. In all, seven sires were crossed with females across four haplotypes; 46 sires were crossed with females from three haplotypes; 146 sires were crossed with females from two haplotypes; and 277 sires were crossed with females from one haplotype (Additional file 4: Table S3). Therefore, a large proportion of sires were joined with dams from more than one haplotype. Additional file 2: Table S2 demonstrates that no one haplotype was indicative of one particular breed and that one haplotype was present in several breeds and hybrid breeds. Haplotype A covered 15 of the lines investigated whilst haplotypes B, C, D and E covered 10, 11, 4 and 10 lines, respectively. No single line was covered by all haplotypes, but the majority was covered by either 3 or 4 haplotypes clearly indicating that haplotype was not breed specific.

We firstly assessed whether there was a relationship between mtDNA haplotype and each of the categories described above irrespective of gender. When we assessed fat density, there were significant statistical differences amongst each of the haplotypes (range *P* < 0.05 to *P* < 0.0001) with haplotype A exhibiting the highest fat density and haplotype D the lowest (Fig. 1a). For muscle depth, haplotype A had the greatest muscle depth and haplotype D the lowest (*P* < 0.0001; Fig. 1b) with haplotype D being more frequently different to the other haplotypes than any of the other haplotypes (range *P* < 0.001 to *P* < 0.0001). To determine fat to leanness ratios, we expressed fat density against muscle depth. Haplotypes A and C represented the highest ratios and haplotypes D and E the lowest (*P* < 0.0001 in each case; Fig. 1c). For lifetime daily gain, haplotype C exhibited the highest gain with differences amongst each of the haplotypes (range *P* < 0.05 to *P* < 0.0001; Fig. 1d). The assessment of teat quality demonstrated differences amongst each of the haplotypes (range *P* < 0.05 to *P* < 0.0001) with haplotype B and C recording the highest and lowest number of good quality teats, respectively (*P* < 0.0001; Fig. 1e). Through the visual assessment of muscle score, we observed that haplotype E had the highest rating and it alone was different to each of the other haplotypes (range *P* < 0.01 to *P* < 0.0001; Fig. 1f). Whilst visual assessment of front legs revealed no differences amongst the haplotypes (Fig. 1g), haplotype D had the highest rating for rear legs but was only significantly different to haplotype E (*P* < 0.05; Fig. 1h).

As there are phenotypic differences between males and females, we assessed pigs from each sex independently for each trait. When assessing females, we observed differences in fat density amongst each of the haplotypes with haplotype A maintaining the greatest density and haplotypes D and E the lowest (range *P* < 0.05 to *P* < 0.0001; Fig. 2a). Haplotype A was also more frequently different to the other haplotypes than the other haplotypes to each other. Muscle depth was greater for haplotypes A and B than the other groupings, with haplotype D being the lowest and the only haplotype significantly different to the other haplotypes (range *P* < 0.05 to *P* < 0.0001; Fig. 2b). When we assessed fat to leanness ratios, haplotypes A and C had higher ratios than the other three haplotypes (range *P* < 0.01 to *P* < 0.0001; Fig. 2c). For lifetime daily gain, haplotype C presented with the highest gain in weight per day, with differences amongst each of the haplotypes (range *P* < 0.01 to *P* < 0.0001; Fig. 2d). There were differences for teat quality with haplotype B having the highest number of good teats and haplotype C the lowest (*P* < 0.0001; Fig. 2e). However, for muscle score, there were no differences amongst each of the groups, indicating that haplotype did not influence muscle score for females (Fig. 2f). Whilst there were no differences amongst the haplotypes for front leg score (Fig. 2g), there were differences for rear legs (Fig. 2h) with haplotype D being higher than haplotype A (*P* < 0.05), B (*P* < 0.05) and E (*P* < 0.01).

**Fig. 3:**
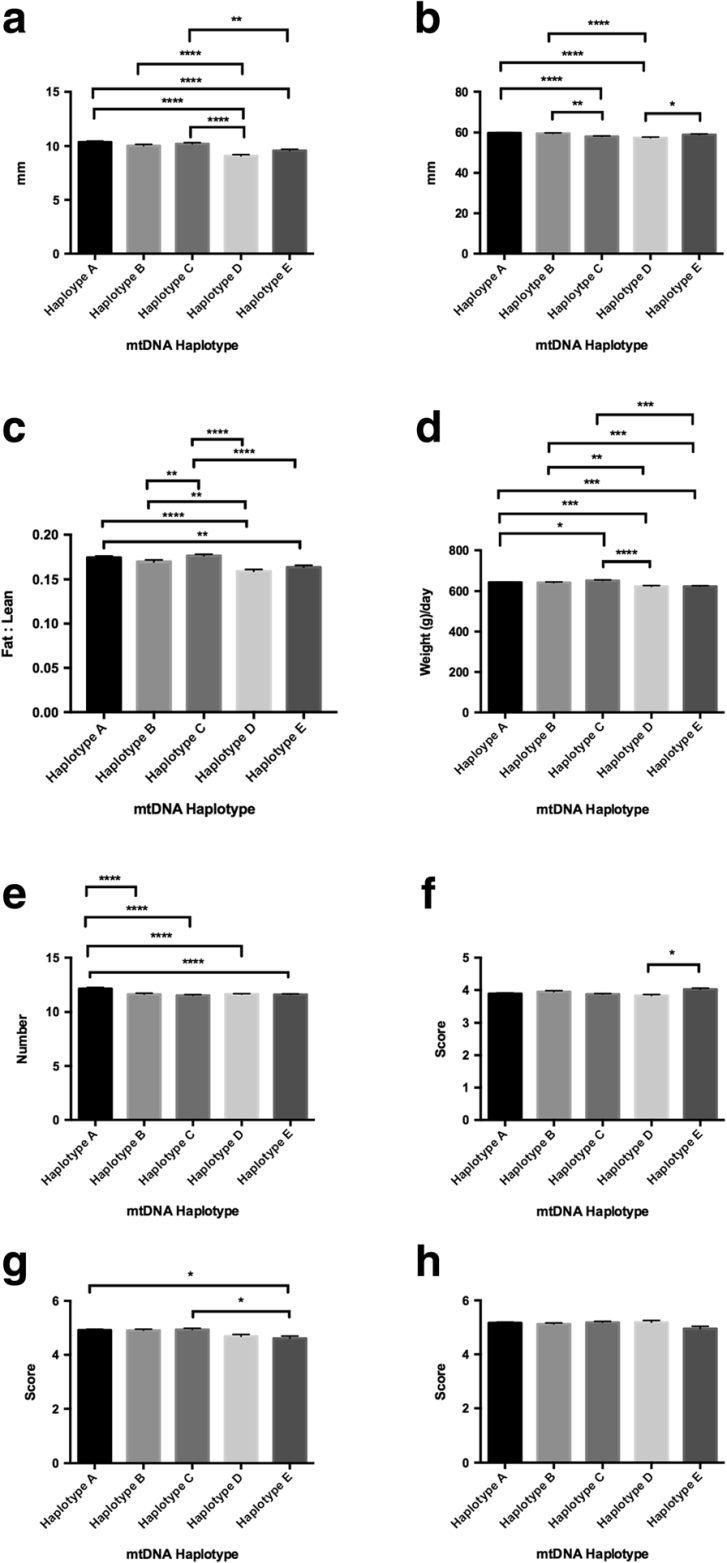
Assessment of phenotypic traits relative to mtDNA haplotype for all males investigated. Graphs represent mean ± SEM values for (**a**) fat density; (**b**) muscle depth; (**c**) fat to leanness ratios; (**d**) lifetime daily gain; (**e**) good teat quality; (**f**) muscle score; (**g**) front leg score; and (**h**) rear leg score. * = *P* < 0.05; ** = *P* < 0.01; *** = *P* < 0.001; **** = *P* < 0.0001

The male offspring exhibited differences across the haplotypes for nearly all of the traits with some different patterns of distribution to the females. For fat density, haplotype A had the greatest depth and haplotype D the lowest with significant differences primarily between these two haplotypes and the other haplotypes (*P* < 0.0001; Fig. 3a). For muscle depth, haplotype A showed the greatest density whilst haplotype D exhibited the least (*P* < 0.0001; Fig. 3b). As for fat density and muscle depth, haplotypes A and D were different from the other haplotypes for fat to leanness ratios (range *P* < 0.01 to *P* < 0.0001; Fig. 3c). For lifetime daily gain, there were a number of differences amongst each of the haplotypes with haplotype C exhibiting the highest lifetime daily gain and haplotype D the lowest (*P* < 0.0001; Fig. 3d). For teat scores, haplotype A had the highest number of quality teats compared with the other haplotypes (each at *P* < 0.0001; Fig. 3e) with haplotype C exhibiting the lowest number. For muscle score, there were only differences between haplotypes D and E, which presented with the lowest and the highest scores, respectively (*P* < 0.05; Fig. 3f). For front legs, there were differences between haplotype E and haplotypes A and C (both *P* < 0.05; Fig. 3g) whilst there were no differences for rear legs (*P* > 0.05; Fig. 3h).

Overall, there were similarities between the males and females in terms of fat and meat density and lifetime daily gain. For fat and meat density, haplotype A appeared to be the dominant haplotype and haplotype D the least dominant. However, when fat and leanness values were expressed as a ratio, haplotypes A and C were the most dominant and D and E the least. Nevertheless, haplotype C was the most dominant for lifetime daily gain and the least for teat quality. Interestingly, the differences in front and rear leg scores were interchangeable between the male and the female populations.

As we observed differences between males and females (Figs. 2 and 3), and it is evident that males had been across females from one or more haplotypes (Additional file 4: Table S3), we further analysed the male and female populations by subdividing them into: i) those generated by sires that had crossed females from one haplotype only; and ii) those which had been generated by sires that had been across two, three or four haplotypes. None of the males had been across all five haplotypes.

**Fig. 4:**
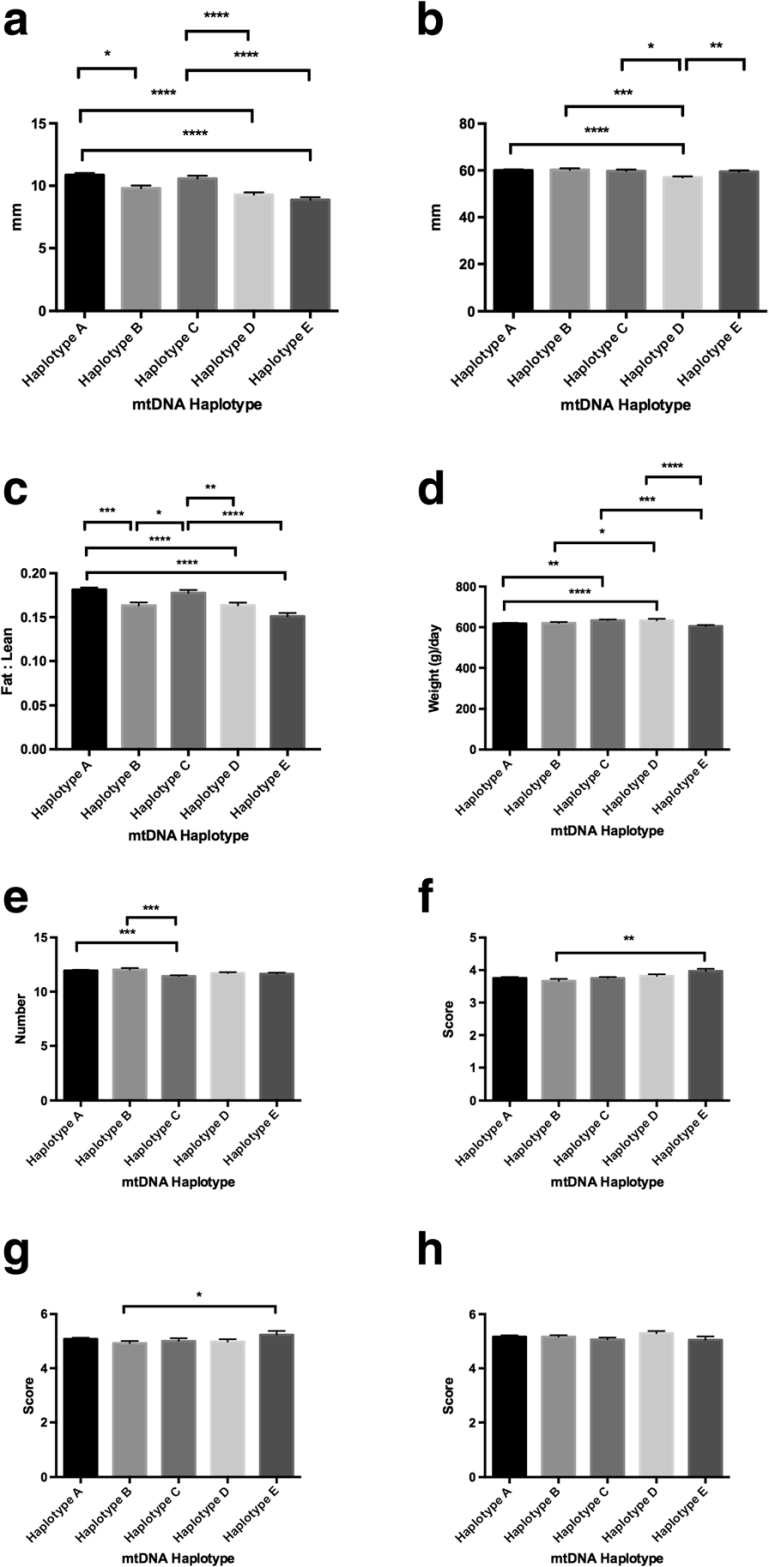
Assessment of phenotypic traits relative to mtDNA haplotype for females where sires were restricted to females from one mtDNA haplotype only at joining. Graphs represent mean ± SEM values for (**a**) fat density; (**b**) muscle depth; (**c**) fat to leanness ratios; (**d**) lifetime daily gain; (**e**) good teat quality; (**f**) muscle score; (**g**) front leg score; and (**h**) rear leg score. * = *P* < 0.05; ** = *P* < 0.01; *** = *P* < 0.001; **** = *P* < 0.0001

When we assessed females that had been generated by sires that had been across only one haplotype, we further observed that there were differences amongst the haplotypes for fat density (range *P* < 0.05 to *P* < 0.0001; Fig. 4a) with haplotype A being predominant, and muscle density (range *P* < 0.05 to P < 0.0001; Fig. 4b) with haplotypes A and B being predominant. Again, haplotype D had the lowest performances for both categories (Fig. 4a - *P* > 0.0001; and Fig. 4b - range *P* < 0.05 to P0.0001). When fat to leanness ratios were assessed, once more haplotypes A and C had the highest fat content whilst haplotypes D and E recorded the lowest (range *P* < 0.05 to *P* < 0.0001; Fig. 4c). Furthermore, there were differences for lifetime daily gain, with haplotype C being most predominant (range *P* < 0.01 to *P* < 0.001; Fig. 4d), but least dominant for teat score (*P* < 0.001; haplotype B most predominant; Fig. 4e). Haplotype E was most predominant for muscle (*P* < 0.01 with haplotype B; Fig. 4f) and front leg (*P* < 0.05 with haplotype B; Fig. 4g) scores but there were no differences for rear legs (Fig. 4h).

**Fig. 5:**
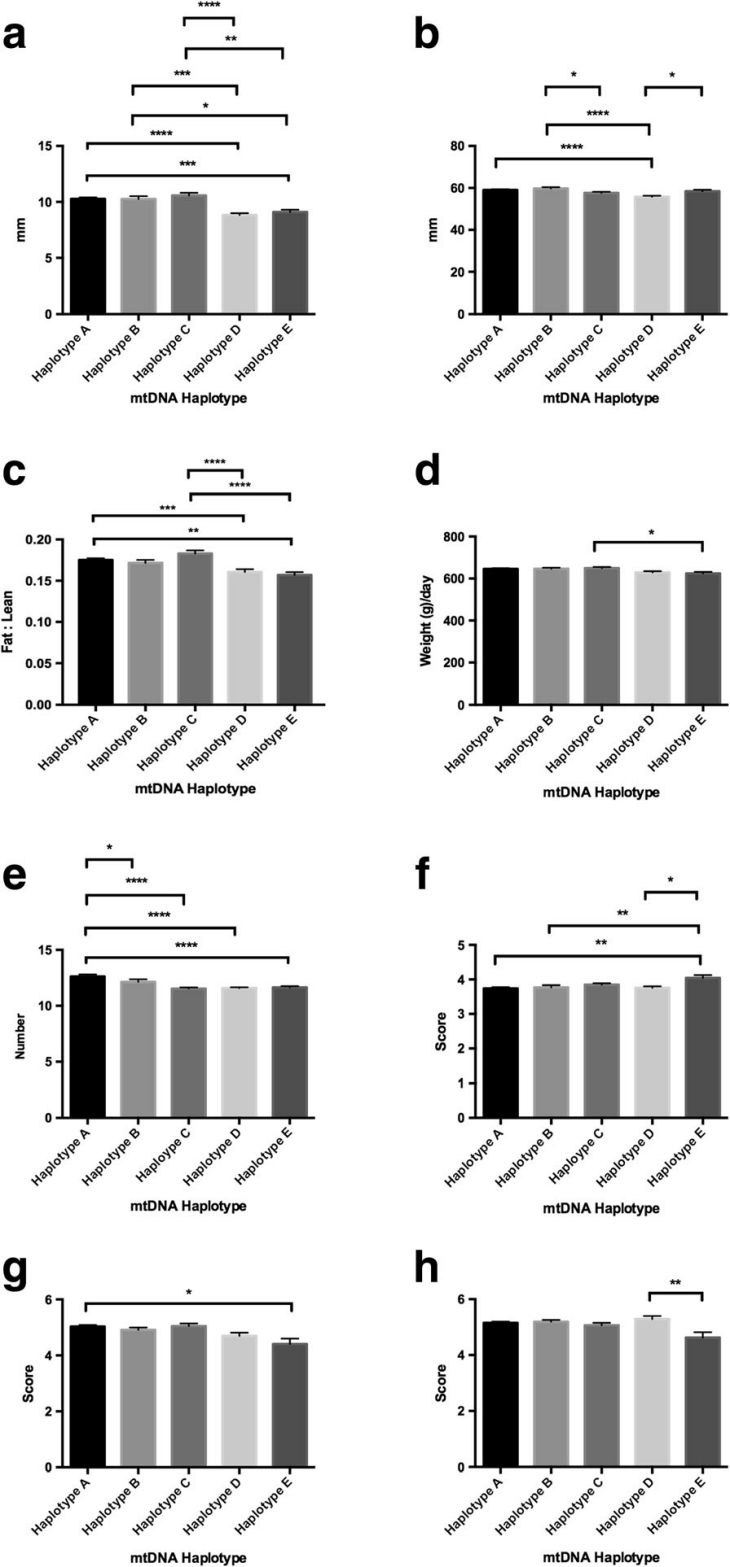
Assessment of phenotypic traits relative to mtDNA haplotype for males where sires were restricted to females from one mtDNA haplotype only at joining. Graphs represent mean ± SEM values for (**a**) fat density; (**b**) muscle depth; (**c**) fat to leanness ratios; (**d**) lifetime daily gain; (**e**) good teat quality; (**f**) muscle score; (**g**) front leg score; and (**h**) rear leg score. * = *P* < 0.05; ** = *P* < 0.01; *** = *P* < 0.001; **** = *P* < 0.0001

When we analysed those males, which had been generated from sires that had only been across one population of females, we once more observed differences in terms of fat density (range *P* < 0.05 to *P* < 0.0001; Fig. 5a), muscle depth (range *P* < 0.05 to *P* < 0.0001), fat to leanness ratios (range *P* < 0.01 to *P* < 0.0001; Fig. 5c), and lifetime daily gain (*P* < 0.05 between haplotypes C and E only; Fig. 5d), which, in this case, showed fewer statistical differences than for the analysis from all animals (Fig. 1d) and the total female (Fig. 2d) and male (Fig. 3d) populations. Teat size (Fig. 5e) was unaffected when compared with all males (cf Fig. 3e) and we saw a predominant effect from haplotype E on muscle (range *P* < 0.05 to *P* < 0.01; Fig. 5f), front leg (*P* < 0.05 with haplotype A only; Fig. 5g) and rear leg (*P* < 0.01with haplotype D; Fig. 5h) scores. Once more, haplotype D was least efficient in terms of fat density (range *P* < 0.001 to *P* < 0.0001; Fig. 5a) and muscle depth (range *P* < 0.05 to *P* < 0.0001; Fig. 5b), whilst haplotype A and C had the highest fat to leanness ratios (range *P* < 0.01 to *P* < 0.0001; Fig. 5c). Haplotype C was the most efficient in terms of life time daily gain (*P* < 0.05 with haplotype E; Fig. 5d) and had the lowest number of good quality teats (*P* < 0.0001 with haplotype A; Fig. 5e) compared with the other haplotypes.

**Fig. 6:**
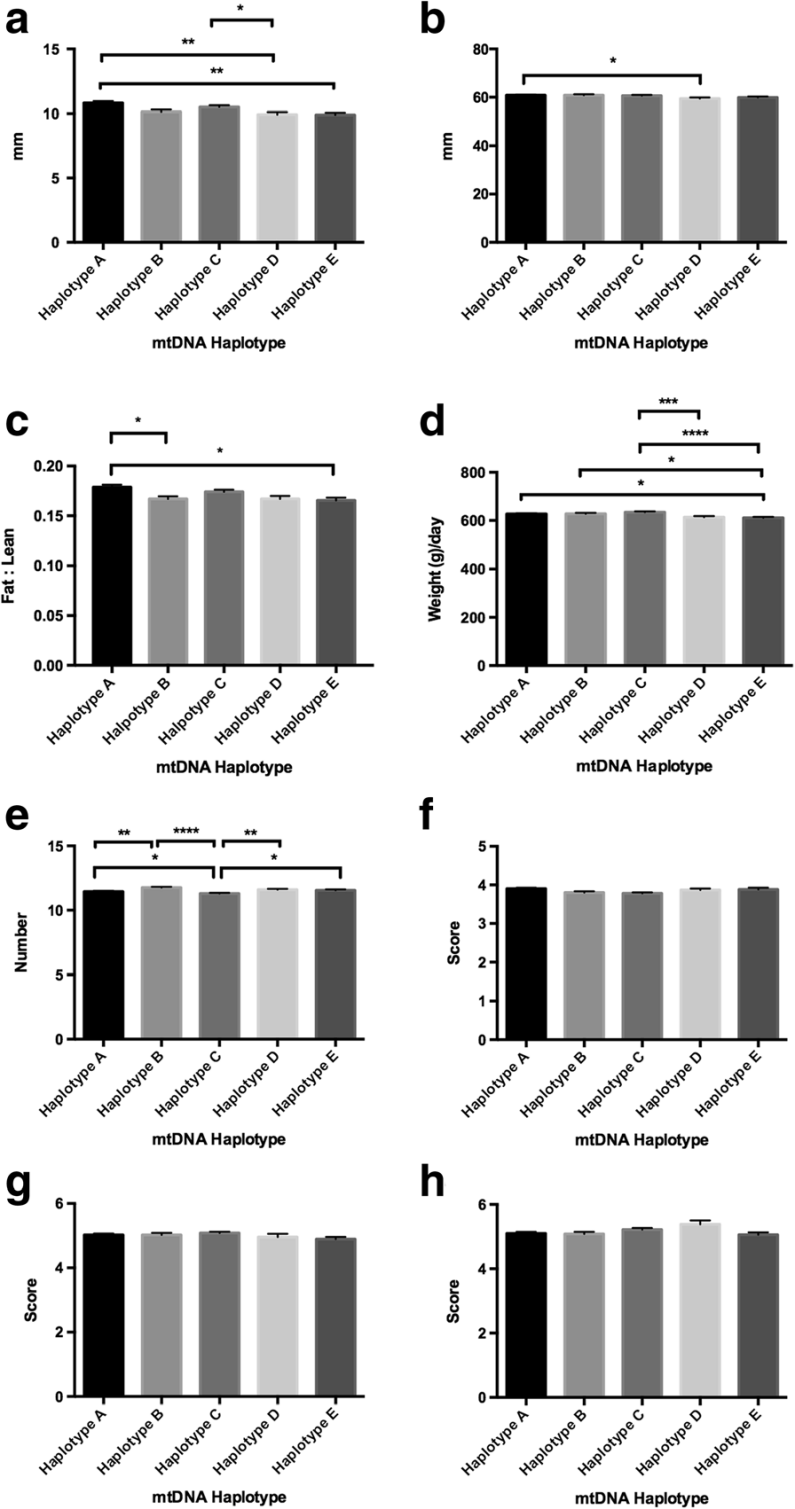
Assessment of phenotypic traits relative to mtDNA haplotype for females where sires were joined to females from more than one mtDNA haplotype. Graphs represent mean ± SEM values for (**a**) fat density; (**b**) muscle depth; (**c**) fat to leanness ratios; (**d**) lifetime daily gain; (**e**) good teat quality; (**f**) muscle score; (**g**) front leg score; and (**h**) rear leg score. * = *P* < 0.05; ** = *P* < 0.01; *** = *P* < 0.001; **** = *P* < 0.0001

By assessing females that had been generated by sires that had been across two, three or four haplotypes (Fig. 6), we further noted differences amongst the haplotypes for fat (between haplotype A and haplotypes D and E - *P* < 0.01, and between haplotypes C and D - *P* < 0.05; Fig. 6a) and meat density (*P* < 0.05 between haplotypes A and D; Fig. 6b), fat to leanness ratios (*P* < 0.05 between haplotypes A and B and E; Fig. 6c), lifetime daily gain (range *P* < 0.05 to *P* < 0.0001; Fig. 6d) and teat quality (range *P* < 0.05 to *P* < 0.0001; Fig. 6e). However, there were no differences for muscle (Fig. 6f), and front (Fig. 6g) or rear (Fig. 6h) leg scores. Interestingly, haplotype A maintained its significant effects on fat density over haplotypes D and E whilst haplotypes C and D were also significantly different to each other (cf Fig. 2a). In terms of meat density, haplotype A was once more different with respect to haplotype D (cf Fig. 2b). However, for fat to leanness ratios, haplotype A exerted significant effects over B and E (*P* < 0.05), as was the case in Figs. 2c (all females) and 4C (sires across only females from one haplotype). The large differences observed for lifetime daily gain, where haplotype E showed the most statistical differences and scored the lowest compared to each of the other haplotypes, and haplotype C was highest (*P* < 0.0001), were maintained (cf Fig. 2d). However, haplotype B maintained its better teat quality scores over the other haplotypes (cf Fig. 2e) and haplotype C maintained a significantly lower score across all three tests (cf Figs. 2e, 4e and 6e).

**Fig. 7:**
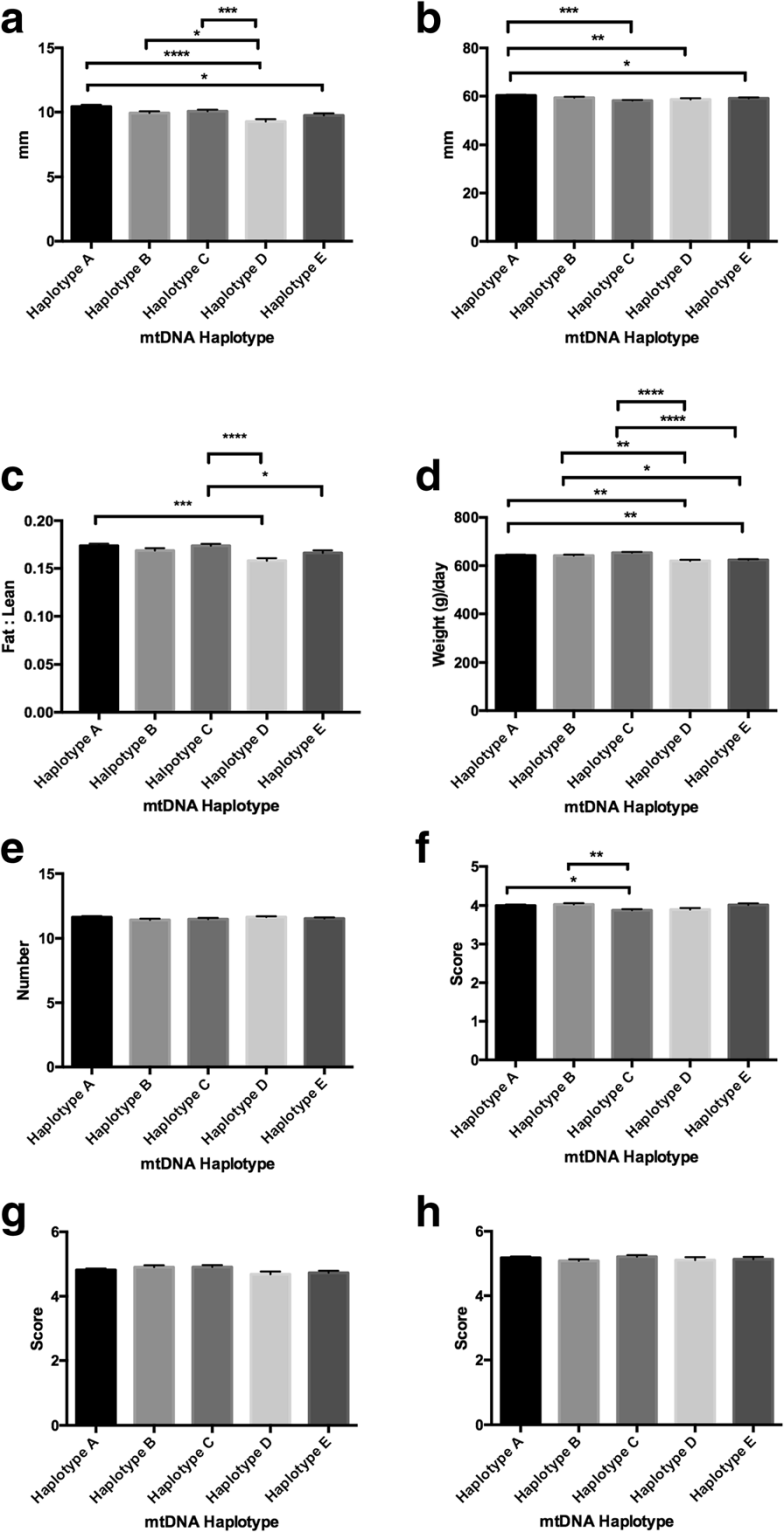
Assessment of phenotypic traits relative to mtDNA haplotype for males where sires were joined to females from more than one mtDNA haplotype. Graphs represent mean ± SEM values for (**a**) fat density; (**b**) muscle depth; (**c**) fat to leanness ratios; (**d**) lifetime daily gain; (**e**) good teat quality; (**f**) muscle score; (**g**) front leg score; and (**h**) rear leg score. * = *P* < 0.05; ** = *P* < 0.01; *** = *P* < 0.001; **** = *P* < 0.0001

When we extended our analyses to male populations that had been generated from sires that had been across females from multiple haplotypes, we saw that the effects on fat density (range *P* < 0.05 to *P* < 0.0001; Fig. 7a) and muscle depth (Haplotype A only with C – *P* < 0.001, D – *P* < 0.01, E – *P* < 0.05; Fig. 7b) were maintained with haplotype A having the highest outcomes (cf Fig. 3a and b, respectively), as was the case with haplotype C for lifetime daily gain (*P* < 0.0001; Fig. 7d; cf. Fig. 3d). Whilst haplotype D continued to have the lowest fat density (range *P* < 0.05 to *P* < 0.0001; Fig. 7a), haplotypes C and D performed least well for muscle depth (range *P* < 0.01 to *P* < 0.001; Fig. 7b). Overall, for the cohorts that had been generated from sires that had been across multiple haplotypes, the effects on fat and meat density tended to be slightly greater for males than for females (cf Figs. 6a and 7a; and 6b and 7b, respectively). Nevertheless, when fat and leanness were assessed as a ratio, haplotypes A and C had the highest levels whilst D was the lowest (range *P* < 0.05 to *P* < 0.0001; Fig. 7c). Although teats (Fig. 7e), front (Fig. 7g) and rear (Fig. 7h) legs were not affected in males, it was evident that there was some effect on muscle score with haplotype C exhibiting the lowest score (Fig. 7f) and was different to haplotypes A (*P* < 0.05) and B (*P* < 0.01).

**Fig. 8:**
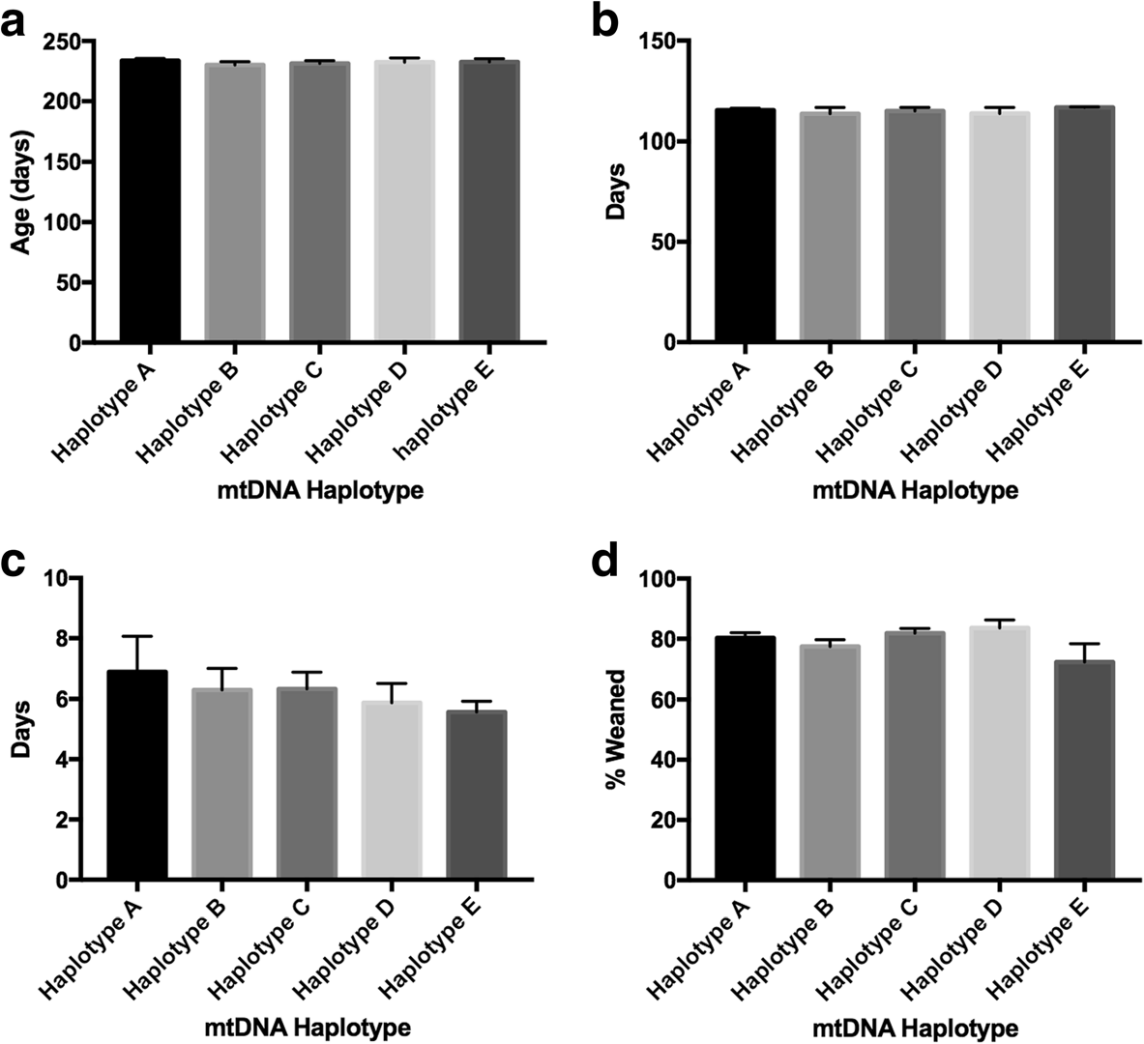
Assessment of phenotypic traits relative to mtDNA haplotype for all animals investigated. Graphs represent mean ± SEM values for (**a**) age of gilt at selection; (**b**) gestational length; (**c**) length of the weaning to oestrus interval; and (**d**) percentage of offspring weaned. No significant differences were found in each of the tests amongst the mtDNA haplotypes

We further investigated whether each of the haplotypes had a predisposition to enhance the age of the gilt at selection (Fig. 8a); gestational length (Fig. 8b); the length of the weaning to oestrus interval (Fig. 8c); and the percentage of offspring weaned (Fig. 8d). In each case, we found that there were no differences between each of the haplotypes (*P* > 0.05).

## Discussion

Mutations in the mitochondrial genome have evolved over generations through a maternal line and become fixed to give rise to mtDNA haplotypes. Whilst human mtDNA mutations are associated with a predisposition to or provide protection against a number of diseases, such as diabetes (type II) [5], cancer [4] and Alzheimer’s [6] and Parkinson’s [7], the effects of mtDNA haplotypes on the phenotype of domestic animals have undergone limited investigation. Here, we have demonstrated in Australian swine that mtDNA haplotypes directly confer both positive and negative effects on the phenotypic traits of a cohort of pigs harbouring different mtDNA haplotypes. Importantly, these haplotypes are not indicative of a specific breed but range across several pure- and crossbreeds. Indeed, Australian commercial pigs are a useful model to study such associations as they have been in relative isolation for a number of years due to import restrictions on new pig lines that protect Australia’s biodiversity (http://www.agriculture.gov.au/import/online-services/bicon) and thus allow interrogation of mtDNA haplotypes over multiple generations of a controlled population.

Specifically, we have seen that mtDNA haplotypes influence fat density, muscle depth, fat to leanness ratios, lifetime daily gain and teat quality. In terms of fat density, haplotype A exhibited the greatest density apart from male offspring that had been generated through the crossing of sires with females from only one haplotype. However, in all cases, haplotype D had the lowest fat density. A similar pattern was observed for muscle depth although there was some interchangeability between haplotypes A and B for greatest depth. Interestingly, when fat to leanness ratios were assessed, it was evident that haplotypes A and C tended to have the highest ratios for fat with haplotypes D and E the lowest. Across all the tests, it is apparent that haplotype A is the most affected as it is the only affected haplotype when females arising from sires that crossed multiple haplotypes are tested. In terms of lifetime daily gain, haplotype C was predominant irrespective of gender and whether sires had been across sows from one, or more than one haplotype. Likewise, haplotype C scored lowest in terms of teat quality for all categories tested, except for male offspring that had been generated from sires that had been across females from more than one haplotype. The importance of teat quality is more likely to lie with females as one of their roles is to suckle their litters, and, for each of the tests on female offspring, haplotype B exhibited the best teat quality. Interestingly, for other supposed female driven phenotypes, such as gestational length, percentage offspring weaned, age of gilt at selection, and weaning oestrus intervals, there were no effects. Consequently, our results indicate evident associations between certain phenotypic traits and mtDNA haplotypes.

With current breeding approaches, where extensive intra- and inter-breed crossing takes place, an animal’s mtDNA haplotype is no longer indicative of its breed but rather defines its common maternal ancestral origins, as indicated in a previous report on pigs and cattle [16, 27]. To this extent, within the population of pigs we analysed, the only consistently shared genetic content amongst each of the animals is likely to be its mtDNA content, which is recycled and passed through the oocytes of maternal relatives from one generation to the next and cannot be altered [28]. This is certainly the case following non-invasive assisted reproductive technologies, such as assisted insemination and in vitro fertilisation [29]. Indeed, following natural conception and assisted reproduction, sperm mtDNA is eliminated prior to fertilisation [30] or in the embryo prior to embryonic genome activation [29]. However, it can be altered by more invasive technologies such as somatic cell nuclear transfer where a donor cell is transplanted into a recipient oocyte harbouring a different population of mtDNA [31]. Nevertheless, the effects of the chromosomal genes are likely to be diluted after each round of breeding unless a paternal trait is pushed through the sire line by, for example, assisted insemination. However, this would still result in the persistence of the respective ancestral mtDNA haplotypes where, for example, two animals may have very similar chromosomal genetic traits but have different mtDNA haplotypes, indicative of their original maternal founder origins. Consequently, the potential for the mitochondrial genome to influence an individual’s performance is high.

In cellular model systems, it has been demonstrated that the mitochondrial genome can influence performance by modulating chromosomal gene expression patterns and metabolic profiles. In studies using reconstructed pig cells, where the mtDNA haplotypes have been replaced, it is evident that the mtDNA content of these reconstructed cells influences respiration and ATP production rates and efficiencies [20] thus modulating cellular bioenergetics. Similar outcomes have been observed in mouse cell models [32, 33] and, more recently, in mice where the mtDNA haplotype induced an aging phenotype [34]. Likewise, embryonic stem cell lines harbouring the same chromosomal genotypes but different mtDNA haplotypes showed that, in their undifferentiated and differentiated states, chromosomal gene expression patterns were modulated based solely on their mtDNA haplotypes [19]. This influences the patterns of neural differentiation and the ability of each of the cell lines to form beating cardiomyocytes. Furthermore, the different mtDNA haplotypes modulated DNA methylation patterns that likely led to the changes in chromosomal gene expression patterns [19, 35]. Indeed, recent studies have demonstrated that metabolites from the citric acid cycle are modulators of histone acetylation [36] and DNA methylation [35], which in turn, can regulate chromosomal gene expression patterns. It is, therefore, likely that the degree of nucleo-mitochondrial compatibility will influence the extent to which the electron transfer chain is used to generate ATP [20, 32, 33], as the electron transfer chain is encoded by both genomes. Consequently, poorer levels of compatibility will likely lead to the build up of metabolites in the citric acid cycle, which produces electron donors for the electron transfer chain, and result in modulated patterns of histone acetylation [36] and DNA methylation [19, 35].

Furthermore, our previous study has shown an association between mtDNA haplotypes and litter size in the same cohort of Australian commercial pigs analysed here and that each of the five haplotypes exhibited different reproductive strategies in order to achieve their respective litter sizes [16]. These included differential developmental competence for maturing oocytes, fertilisation rates and development to the blastocyst stage, the final stage of preimplantation embryo development. Interestingly, our results show that pigs from haplotype A are more predisposed to having a higher fat density, muscle depth and fat to leanness ratios than pigs from the other haplotypes, which would be indicative of preferred meat quality. On the other hand, haplotype C pigs were more predisposed to having higher lifetime daily gain and, in some tests, higher fat to leanness ratios and haplotype B better teat quality. However, haplotypes A and B exhibited lower litter sizes than haplotypes C, D and E with haplotypes C and D showing increased reproductive capacity [16]. Consequently, there appears to be a trade off in traits [37, 38] between, for example, reproductive capacity and fat, muscle density and fat to leanness ratios, especially in the case of haplotype A. Indeed, ‘trade off’ has been reported in the context of reproductive capacity and energetic expense in birds [39], where females investing in larger oocytes and, thus, making a higher maternal investment had larger reproductive organs, and, as a consequence, an overall larger body mass with a higher resting metabolic rate [39]. Further evidence comes from cattle where females with the best estimated breeding values are leaders for growth and carcass traits. However, they often have poor fertility [40–42]. Interestingly, cattle mtDNA appears to alter the phenotype of bison in that bison with cattle mtDNA are consistently smaller in both nutritionally poor and nutritionally rich environments [13]. Moreover, in humans, a mtDNA pathogenic mutation is highly enriched in Tibetan highlanders, which has likely required this population to adapt to hypoxic conditions at the expense of oxidative phosphorylation [43]. Therefore, mtDNA plays an important role in determining phenotypes, however, its effects are limited to the development of certain traits. The current work illustrates that it could be beneficial for pig and livestock breeders to identify the mtDNA haplotypes of their breeding populations, in order to make informed decisions on whether to continue to enhance certain traits or to rescue inferior traits in certain animals.

## Conclusions

In all, our study illustrates that mtDNA haplotypes confer positive advantages to phenotypes, such as fat density, muscle depth, fat to leanness ratios, lifetime daily gain and teat quality in pigs. Moreover, there appears to be a trade-off between reproductive capacity and other traits, such as meat quality, as demonstrated in mtDNA haplotype A. Whilst this is not unusual, it highlights why specific animals are maintained in breeding programs as they provide traits that ensure profitability but at a cost to other traits. The evaluation of mtDNA haplotypes in breeding livestock and using this additional information to alter breeding strategies could add significantly to an animal’s estimated breeding value and thus provide further improvement to certain domestic animal populations.

## Additional files

Additional file 1: **Table S1.** Number of animals analysed per mtDNA haplotype. (DOCX 47 kb)

Additional file 2: **Table S2.** Distribution of breeds covering each haplotype. (DOCX 91 kb)

Additional file 3: **Figure S1.** Molecular Phylogenetic analysis by Maximum Likelihood method. Time of divergence was estimated using the RelTime method and based on the Asian European split of 750,000 YBP. The estimated divergence time for mtDNA haplotypes A and B was 50,000 YBP; A and C 90,000 YBP; A and D 750,000 YBP; and D and E 50,000 YBP. (DOCX 52 kb)

Additional file 4: **Table S3.** Distribution of sires across haplotypes. (DOCX 29 kb)

## Abbreviations

BIC: Bayesian Information Criterion
HV: hypervariable
MCL: Maximum Composite Likelihood.
mtDNA: mitochondrial genome / mitochondrial DNA
