# Supplementary material for: The association of mitochondrial DNA haplotypes and phenotypic traits in pigs
Source: BMC Genet. 2018 Jul 6;19:41. doi: 10.1186/s12863-018-0629-4 (PMC6035439; doi:10.1186/s12863-018-0629-4)
Supplement: Supplementary file 1 — Table S1. Number of animals analysed per mtDNA haplotype. (DOCX 47 kb) [file 12863_2018_629_MOESM1_ESM.docx]

**Table S1: Number of animals analysed per mtDNA haplotype**

| Haplotype | No. pigs | Females | Males | Sires across haplotype |
| --- | --- | --- | --- | --- |
| A | 1742 | 811 | 931 | 250 |
| B | 991 | 452 | 539 | 132 |
| C | 1338 | 674 | 664 | 143 |
| D | 840 | 445 | 395 | 86 |
| E | 776 | 380 | 396 | 116 |

Animals were assigned to their relevant mtDNA haplotypes and subdivided based on gender. Sires across haplotype refers to the number of sires that were joined to females to produce the progeny analysed.
