# Supplementary material for: The association of mitochondrial DNA haplotypes and phenotypic traits in pigs
Source: BMC Genet. 2018 Jul 6;19:41. doi: 10.1186/s12863-018-0629-4 (PMC6035439; doi:10.1186/s12863-018-0629-4)
Supplement: Supplementary file 2 — Table S2. Distribution of breeds covering each haplotype. (DOCX 91 kb) [file 12863_2018_629_MOESM2_ESM.docx]

|  | **Haplotype** | | | | |
| --- | --- | --- | --- | --- | --- |
| **Breed** | **A**  **(%)** | **B**  **(%)** | **C**  **(%)** | **D**  **(%)** | **E**  **(%)** |
| Landrace | 3.7 | 0.0 | 0.0 | 84.7 | 43.8 |
| Large White | 15.5 | 38.9 | 25.8 | 0.0 | 0.0 |
| Large White Sire line | 4.6 | 31.2 | 28.2 | 0.0 | 2.3 |
| Hybrid large white and landrace | 4.1 | 0.4 | 1.5 | 0.0 | 0.0 |
| Duroc Sire Line | 38.5 | 6.9 | 26.1 | 0.0 | 9.6 |
| Duroc Sow Line | 12.8 | 0.7 | 11.8 | 0.0 | 7.1 |
| Hybrid Large White and Duroc | 0.3 | 0.0 | 0.0 | 0.0 | 0.0 |
| Landrace based sire line | 10.2 | 12.4 | 0.0 | 14.4 | 26.9 |
| Duroc and Large White | 0.2 | 1.9 | 0.6 | 0.0 | 0.0 |
| Landrace and Large White | 0.0 | 0.0 | 0.1 | 0.0 | 0.0 |
| Large White and Landrace | 0.1 | 0.0 | 0.3 | 0.0 | 0.5 |
| Landrace and Duroc | 4.4 | 1.3 | 3.8 | 0.0 | 2.3 |
| Large White and Duroc | 0.1 | 0.0 | 0.0 | 0.4 | 0.0 |
| Landrace and Duroc | 0.1 | 0.0 | 0.0 | 0.4 | 0.0 |
| Landrace, Duroc Large White | 0.2 | 0.1 | 0.4 | 0.1 | 0.0 |
| Large White, Duroc and Landrace | 0.0 | 0.2 | 0.4 | 0.0 | 0.8 |
| Large White and Duroc | 0.1 | 0.0 | 0.0 | 0.0 | 0.9 |
| Duroc and Landrace | 5.3 | 6.1 | 0.8 | 0.0 | 5.8 |
|  | 100 | 100 | 100 | 100 | 100 |

**Table S2. Distribution of breeds covering each haplotype.**
