# Supplementary material for: The association of mitochondrial DNA haplotypes and phenotypic traits in pigs
Source: BMC Genet. 2018 Jul 6;19:41. doi: 10.1186/s12863-018-0629-4 (PMC6035439; doi:10.1186/s12863-018-0629-4)
Supplement: Supplementary file 3 — Figure S1. Molecular Phylogenetic analysis by Maximum Likelihood method. Time of divergence was estimated using the RelTime method and based on the Asian European split of 750,000 YBP. The estimated divergence time for mtDNA haplotypes A and B was 50,000 YBP; A and C 90,000 YBP; A and D 750,000 YBP; and D and E 50,000 YBP. (DOCX 52 kb) [file 12863_2018_629_MOESM3_ESM.docx]

**Figure S1.** Molecular Phylogenetic analysis by Maximum Likelihood method. Time of divergence was estimated using the RelTime method and based on the Asian European split of 750,000 YBP. The estimated divergence time for mtDNA haplotypes A and B was 50,000 YBP; A and C 90,000 YBP; A and D 750,000 YBP; and D and E 50,000 YBP.
