# Supplementary material for: The association of mitochondrial DNA haplotypes and phenotypic traits in pigs
Source: BMC Genet. 2018 Jul 6;19:41. doi: 10.1186/s12863-018-0629-4 (PMC6035439; doi:10.1186/s12863-018-0629-4)
Supplement: Supplementary file 4 — Table S3. Distribution of sires across haplotypes. (DOCX 29 kb) [file 12863_2018_629_MOESM4_ESM.docx]

**Table S3: Distribution of sires across haplotypes**

| **Number of haplotypes** | | | | |
| --- | --- | --- | --- | --- |
|  | 4 | 3 | 2 | 1 |
| No. sires | 7 | 46 | 146 | 277 |
